# Virtual reality use and patient outcomes in palliative care: A scoping review

**DOI:** 10.1177/20552076231207574

**Published:** 2023-11-01

**Authors:** Mairead Moloney, Owen Doody, Martina O’Reilly, Michael Lucey, Joanne Callinan, Chris Exton, Simon Colreavy, Frances O’Mahony, Pauline Meskell, Alice Coffey

**Affiliations:** 1 Department of Nursing and Midwifery, University of Limerick, Limerick, Ireland; 2 Health Implementation Science and Technology, University of Limerick, Limerick, Ireland; 3 Health Research Institute, University of Limerick, Limerick, Ireland; 4 Milford Care Centre, Limerick, Ireland; 5 Department of Computer Science and Information Systems, University of Limerick, Limerick, Ireland

**Keywords:** Virtual reality, palliative care, patient outcomes, scoping review

## Abstract

**Objective:**

Virtual reality is increasingly used in healthcare settings. Potentially, it's use in palliative carecould have a positive impact; however, there is limited evidence on the scope, purpose and patient outcomes relating to virtual reality use in this context. The objective of this scoping review is to chart the literature on virtual reality use in palliative care, identifying any evidence relating to biopsychosocial patient outcomes which could support its use in practice.

**Methods:**

A scoping review of the literature, involving . a systematic search across 10 electronic bibliographic databases in December 2021, . Eligibility criteria were primary research studies, of any research designwithin a 10-year timeframe, which reported on virtual reality use and patient outcomes in palliative care. A total of 993 papers were identified, andcomprehensive screening resulted in 10 papers for inclusion.

**Results:**

This scoping review identified 10 papers addressing virtual reality in palliative care, published within a three-year timeframe 2019–2021. Research methodologies included mixed methods, quantitative and qualitative. The evidence highlightsvirtual reality use with patients receiving palliative care in a variety of settings, and data around useability, feasibility and acceptability is positive. However, the evidence regarding biopsychosocial patient outcomes linked to virtual reality use is limited.

**Conclusion:**

Virtual reality is gathering momentum in palliative care and is potentially a helpful intervention; however more research is needed to underpin the evidence base supporting its application, particularly in understanding the impact on biopsychosocial patient outcomes and ascertaining the best approach for measuring intervention effectiveness.

## Introduction

Virtual reality (VR) is described as the simulation of reality where the users are immersed in a virtual environment creating an illusion that the environment exists.^1^ It involves technologically generated virtual environments with visual graphics, processing, display and sounds to create an interactive world.^2^ The user becomes immersed in their new visual environment, often accompanied by aural components and other sensory inputs.^3^ In the past decade, the use of VR has become more prevalent with the general public, particularly across the entertainment, education and industry sectors. With technological advancement and expertise development, VR is more accessible and commercially available than ever before.^2–4^ VR use in the healthcare sector is also gathering momentum, and reports of VR applications as direct patient care interventions are becoming more common.^3–5^ For example, VR has been found to have a positive effect on managing the symptoms of depression,^6^ pain management^7–9^ and anxiety management.^10^

This review is particularly focused on the specialist area of palliative care (PC). The World Health Organisation^11^ defines PC as an approach that improves the quality of life of people with life-threatening or life-limiting health conditions; it aims to prevent and relieve suffering (physical, psychological, social and spiritual) through identification, assessment and treatment of pain and other symptoms. Recent systematic reviews have highlighted VR as a helpful treatment modality in PC. Martin et al.^12^ found VR using head-mounted displays (HMDs) in PC to be promising for addressing patient symptoms. Mo et al.,^13^ in a review of quantitative studies, found VR to be feasible and acceptable in PC, and a review by Carmont and McIlfatrick^14^ found VR could support patients, families and healthcare professionals in PC. All recent reviews advocate the need for more research and the need to provide definitive recommendations in this specialist area.^12–14^ However, there remains limited knowledge on how VR use in PC impacts patients’ outcomes. Therefore, a scoping review was deemed an appropriate enquiry for this study, with the objective of charting the literature on VR use in PC, identifying any evidence relating to biopsychosocial patient outcomes which could support its use in practice. While our review corroborates previous reviews in this area,^12–14^ it also offers unique insights on patient outcomes related to VR use, reporting on the type of outcomes measured across the biopsychosocial dimensions, as well as the varied measurement approaches and tools used. In addition, the range of VR technology software/hardware, policies and the different healthcare professionals involved in facilitating VR experiences are reported. This knowledge and evidence are necessary to underpin VR use in PC and may be helpful for researchers who are considering study designs which aim to objectively or subjectively measure patient outcomes in VR use.

## Methods

A scoping review methodology was employed as it is helpful for identifying existing knowledge and evidence on a particular phenomenon, while simultaneously identifying gaps where further research is needed.^15–19^ While VR use in PC is developing and increasing,^2–4^ there remains limited evidence on biopsychosocial patient outcomes linked to VR use in this context; hence, it was considered premature at this stage to conduct a more comprehensive systematic review. This scoping review charts the literature from primary research studies thus providing a foundation and direction for further research on VR use in this context. The review did not aim to systematically appraise the literature or provide definitive answers based on synthesised results; hence, a methodological quality or risk of bias appraisal was not conducted.

The Arksey and O’Malley^15^ scoping review framework guided the review, which was conducted in line with the five-step process: (a) identifying the research question, (b) identifying relevant studies, (c) selecting studies, (d) charting the data and (e) arranging, summarising and communicating the outcomes. Further scoping review advancements by Levac et al.^16^ and guidance from JBI and the JBI Collaboration^17,18^ informed the review methodology. The review steps are sequential; however, the process is iterative, and steps were developed and revisited throughout the research process.

### Research ethics and informed patient consent

As a scoping review, this research did not involve people as participants; therefore, it did not require ethical approval or informed patient consent. The results of this scoping review are reported in accordance with the *Preferred Reporting Items for Systematic Reviews and Meta-Analyses extension for Scoping Reviews* (PRISMA-ScR) (Supplemental File 1).

### Protocol and registration

A protocol was registered with Open Science Framework on 21 December 2021,^21^ and at that time, a preliminary search of PROSPERO, Medline, the Cochrane Database of Systematic Reviews, and JBI Evidence Synthesis was conducted, and no current or in-progress scoping reviews or systematic reviews were identified on VR use and patient outcomes in PC.

### Step 1: Identification of the research question

The research question was defined through discussion and deliberation within the research team who were represented by PC nursing, PC medicine, occupational therapy, computer technology, library/information specialist and nurse academics. Further consultation with researchers and experts in the field of PC and computer science helped further refine concepts and contexts. The research question was structured using the ‘PCC’ mnemonic, which denotes population, concept and context. This mnemonic is useful in scoping reviews for constructing a clear and meaningful research question.^18^ The primary research question is broad, reflecting the core elements of PCC specific to this study, and aligns with the scoping review objective and methodology. To address the primary research question, further elements and dimensions were explored by using subquestions on aspects of VR technology, application, patient outcomes and VR-related guidelines and policies.

#### Primary research question

What knowledge and evidence exists in the literature on VR use in PC, particularly in relation to biopsychosocial patient outcomes?

#### Research subquestions 1–5

1. What types of VR modalities have been used and reported in PC environments?
2. What patient groups has VR been used with, and in what context (e.g. inpatient care or home care), and who was involved in facilitating the VR experience?
3. How have VR interventions been evaluated to date, i.e. what patient outcomes are measured?
4. What evidence base (if any) exists on the clinical effectiveness of VR in PC?
5. What guidelines/policies exist regarding the use of VR in PC?

### Step 2: Identification of relevant studies

#### Search strategy

A preliminary search for existing scoping reviews and/or systematic reviews on VR use and patient outcomes in PC was conducted on 21 December 2021, and no reviews (completed or underway) were identified at that time. The search strategy involved the development of search terms using keywords and medical subject headings (MeSH) (Table 1), with Boolean operators used to combine and expand searches. As recommended in JBI guidance,^18^ a preliminary limited search of two databases (i.e. Cumulative Index to Nursing and Allied Health Literature (CINAHL) and PubMed) was conducted to test and refine the search strategy; however, no new keywords or combinations were identified for the final search. Following this, a systematic search for literature was applied across 10 electronic bibliographic databases: Academic Search Complete, Cochrane Central Register of Controlled Trials (CENTRAL), Cochrane Database of Systematic Reviews, CINAHL, JBI Evidence Synthesis, Medline, PubMed, Embase, Scopus and Psycinfo.

**Table 1.** Search terms.

MeSH		Open synonyms
(MM ‘Terminally Ill’) OR (MM ‘Terminal Care+’) OR (MM ‘Palliative Care’) OR (MM ‘Palliative Medicine’) OR (MM ‘Hospice and Palliative Care Nursing’)	OR	palliative care or terminal care or terminal illness or terminal disease or terminal cancer or end of life or end-of-life or hospice care or supportive care or life limiting illness or life limiting disease or advanced cancer or advanced illness or advanced disease
	AND	
(MM ‘Augmented Reality’) OR (MM ‘Virtual Reality’) OR (MM ‘Virtual Reality Exposure Therapy’)	OR	virtual reality or virtual technolog* or virtual environment or virtual world

#### Inclusion and exclusion criteria

Criteria for inclusion and exclusion of studies were discussed extensively within the research team (Table 2). The population was restricted to adults (≥18 years of age) receiving PC, as adult PC was the focus of this review. The concept was VR and patient outcomes. VR was deliberately broadly defined in order to scope and capture the diverse range of new and existing technological applications (e.g. not limited to HMDs). The context was PC, terminal care, hospice care and/or end-of-life care. Within context, the provision of PC could be on an inpatient or outpatient basis in a variety of healthcare or home settings, i.e. it was not confined to a PC specialist centre. The criteria were tested on a sample of 20 abstracts prior to the review process to ensure that they were sufficiently robust in capturing relevant studies, ensuring exclusion of non-eligible studies.

**Table 2.** Inclusion/exclusion criteria.

Inclusion	Exclusion
Population: adults >18 years of age in receipt of palliative/hospice care Concept: virtual reality and patient outcomes Context: palliative care, terminal care, hospice care, end-of-life care Types of information sources: studies published between 01 Jan 11 and 31 Dec 21. Primary research studies include qualitative, quantitative and mixed method studies but not limited to research designs. Studies published in the English language	Population: participants < 18 years of age Concept: any concept not related to virtual reality Context: any context other than palliative care, terminal care, hospice care, end-of-life care Types of information sources: studies earlier than 2011. Non-primary research-based papers such as editorials, notes, letters, commentaries, discussion papers and opinion pieces. Non-published thesis. Not published in English

#### Types of information sources

Sources of information considered for this review included all types of published primary research studies, qualitative, quantitative and mixed methods, which reported on any aspects of biopsychosocial patient outcomes resulting from VR use in PC. Limits and exclusion criteria were imposed on sources such as editorials, notes, letters, opinion pieces, commentaries and discussion papers. The objective of this scoping review is to chart the literature on VR use in PC, identifying any evidence relating to biopsychosocial patient outcomes which could support its use in practice; therefore, primary research studies which reported on such outcomes were deemed higher order and more appropriate to the review and research question. To refine the search and align with the advancements and resurgence of VR use in the general public,^2–4^ a 10-year timeframe from 2011 to 2021 was applied. Only sources of information in the English language were included as this was the first language of the research team. The 10 papers included in this review were screened using backward and forward citation chaining, where reference lists and citations were screened, and no further papers were identified. In addition, a search for ongoing and unidentified clinical trials was conducted in Google Scholar, ClinicalTrials.gov, EU Clinical Trial Register and the WHO International Clinical Trials Registry Platform search portal. No trials in PC were identified as of 21 December 2021. This process was guided by the PRESS Guideline Evidence-Based Checklist.^19^

### Step 3: Study selection

**Figure 1.**
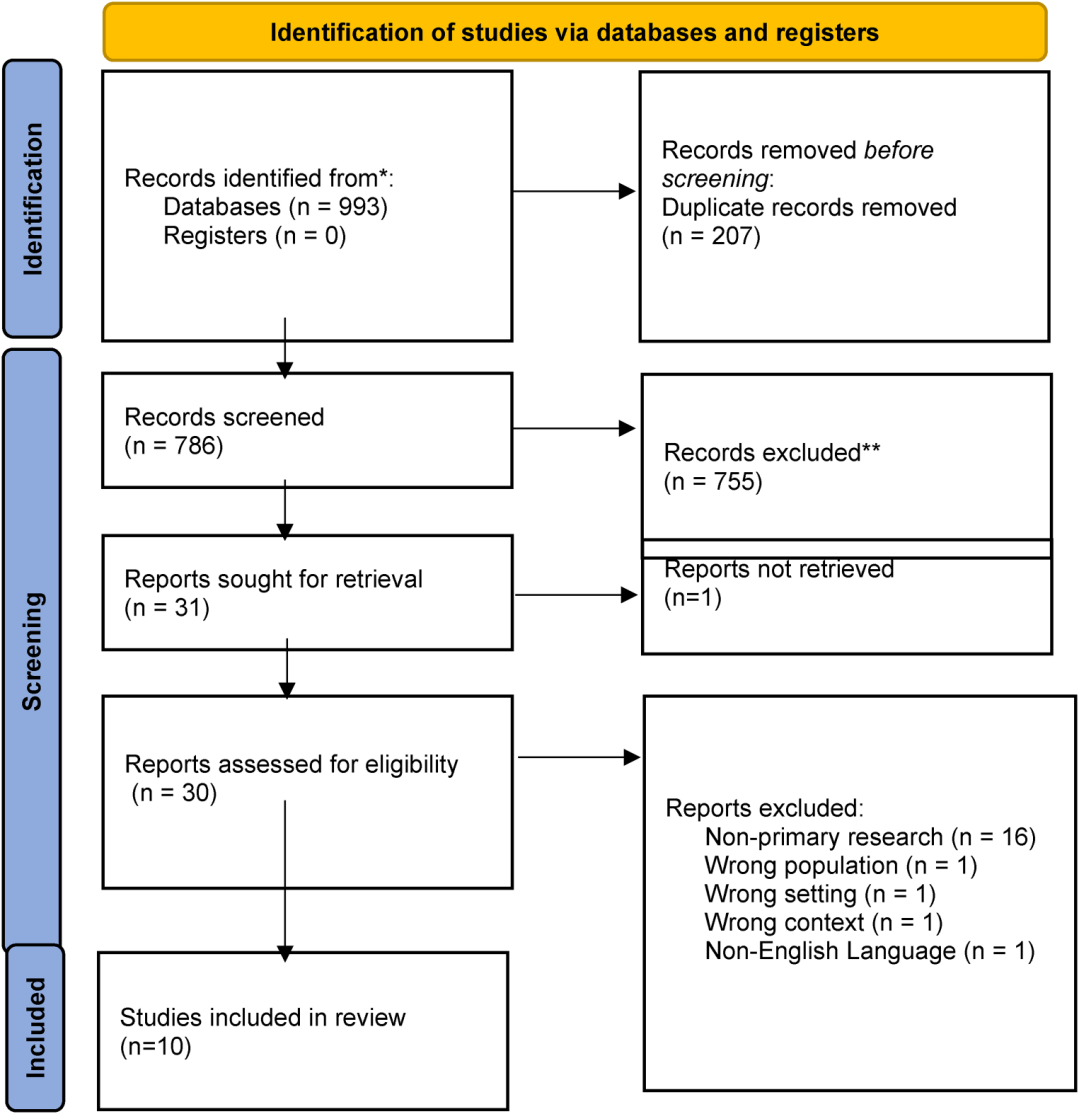
PRISMA flow diagram.^20^

Following the search, all identified records (*N* = 993) were collated and uploaded to Rayyan, an online management resource for systematic reviews, and duplicates were removed (*n* = 207). Titles and abstracts of potentially relevant papers (*n* = 786) were initially screened by two independent reviewers (MM and OD), aligning with the inclusion criteria for the review. Papers that did not meet the inclusion criteria were excluded (n = 755). Full-text review of 31 papers was conducted (MM, OD), and a further 21 papers were excluded with reasons recorded on the PRISMA flow diagram (Figure 1) and in Supplemental File 2. Any disagreements or uncertainties at this stage were resolved through discussion or with the third reviewer (AC) as necessary. Ten papers were included in the review.

### Step 4: Charting the data

In accordance with Arksey and O’Malley's framework,^15^ summaries from each paper were extracted and charted. The charting table was designed by the authors specifically for the review. Pre-testing of the table by the research team resulted in slight changes to the wording and order of the subquestions in the actual study as compared to the protocol. Data charting included summaries from each paper regarding author, year, publication origin, country origin, aim/purpose, methods/methodology, VR description, and then the subsequent table columns representing the review questions.

### Step 5: Arranging, summarising, and communicating the outcomes

Arksey and O’Malley's^15^ final stage arranges, summarises and communicates the outcomes of the review. A description of the methodological characteristics and geographical locations of papers is included hereunder. The review results are collated, summarised, and organised in descriptive form under the five subquestions identified in step 1 of the review. Tables are used to augment the narrative.

## Results

### Description of papers

This scoping review identified 10 papers reporting on VR use and patient outcomes in PC. Table 3 lists the included papers, and Supplemental File 1 is the data charting table. These papers were current and spanned three years 2019–2021, despite the 10-year timeframe limit of the review. The review papers originated from the United States of America (*n* = 5), United Kingdom (*n* = 3), Italy (*n* = 1) and Japan (*n* = 1). Paper methodologies or approaches were varied and included mixed methods (*n* = 3),^22–24^ quantitative (*n* = 4)^25–28^ and qualitative (*n* = 3).^29–31^

**Table 3.** Summary of included studies (full data charting table (Supplemental File 1)).

Author(s), year of publication, origin	Aim/purpose	Methodology	Virtual reality description
Brungardt, A., Wibben, A., Tompkins, A.F., Shanbhag, P., Coats, H., LaGasse, A.B., Boeldt, D., Youngwerth, J., Kutner, J.S. and Lum, H.D. (2021) ‘Virtual reality-based music therapy in palliative care: a pilot implementation trial’, *Journal of Palliative Medicine*, *24*(5), 736–742. United States of America	To evaluate implementation measures of feasibility, usability and acceptability of a VR-based music therapy (MT) intervention	A pilot implementation study of a 2-day VR–MT intervention using mixed methods	VR experience with sound and music Described as an intervention
Dang, M., Noreika, D., Ryu, S., Sima, A., Ashton, H., Ondris, B., Coley, F., Nestler, J. and Fabbro, E.D., (2021) ‘Feasibility of delivering an avatar-facilitated life review intervention for patients with cancer’, *Journal of Palliative Medicine*, *24*(4), 520–526. United States of America	To determine the feasibility of an avatar-based intervention for facilitating life review in patients with advanced cancer	Prospective trial to establish feasibility and acceptability of an avatar-based intervention for facilitating life review in patients with advanced cancer	An avatar-facilitated life review Described as an intervention
Ferguson, C., Shade, M.Y., Blaskewicz Boron, J., Lyden, E. and Manley, N.A. (2020) ‘Virtual reality for therapeutic recreation in dementia hospice care: a feasibility study’, *American Journal of Hospice and Palliative Medicine*, *37*(10), 809–815. United States of America	To explore acceptability, tolerability and subjective experience of virtual reality (VR) as therapeutic recreation for hospice patients living with dementia	A feasibility study using descriptive design	VR experience Described as a therapeutic recreation
Johnson, T., Bauler, L., Vos, D., Hifko, A., Garg, P., Ahmed, M. and Raphelson, M. (2020) ‘Virtual reality use for symptom management in palliative care: a pilot study to assess user perceptions’, *Journal of Palliative Medicine*, *23*(9), 1233–1238. United States of America	To examine the utility of VR for palliative care patients	A prospective single-centre, single-arm study	VR experience Described as an intervention
Lloyd, A. and Haraldsdottir, E. (2021) ‘Virtual reality in hospice: improved patient well-being’, BMJ Support Palliative Care, 11(3), 344–350. Scotland	To trial VR technology and consider what benefits may emerge for hospice inpatients	Qualitative exploratory study	VR session Described as a therapeutic intervention
Moscato, S., Sichi, V., Giannelli, A., Palumbo, P., Ostan, R., Varani, S., Pannuti, R. and Chiari, L. (2021) ‘Virtual reality in home palliative care: brief report on the effect on cancer-related symptomatology’, *Frontiers in Psychology*, 12 (709154). Italy	To assess the effect of an immersive VR-based intervention conducted at home on anxiety, depression and pain over 4 days and to evaluate the short-term effect of VR sessions on cancer-related symptomatology	A mixed methods pre–post-single-arm study	VR-based intervention Described as an Intervention
Niki, K., Okamoto, Y., Maeda, I., Mori, I., Ishii, R., Matsuda, Y., Takagi, T. and Uejima, E. (2019) ‘A novel palliative care approach using virtual reality for improving various symptoms of terminal cancer patients: a preliminary prospective, multicenter study’, *Journal of Palliative Medicine*, *22*(6), 702–707. Japan	To verify whether simulated travel using virtual reality (VR travel) is efficacious in improving symptoms in terminal cancer patients	Prospective, multicentre, single-arm study	Simulated travel using VR Described as an intervention
Nwosu, A.C., Mills, M., Roughneen, S., Stanley, S., Chapman, L. and Mason, S.R. (2021) ‘Virtual reality in specialist palliative care: a feasibility study to enable clinical practice adoption’ *BMJ Supportive & Palliative Care, 0, 1–5.* United Kingdom	To (a) explore the feasibility of implementing VR therapy, for patients and caregivers, in a hospital specialist inpatient palliative care unit and a hospice and (b) identify questions for organisations, to support VR adoption in palliative care	Quality improvement project	VR distraction therapy Described as a distraction therapy
Perna, L., Lund, S., White, N. and Minton, O. (2021) ‘The potential of personalized virtual reality in palliative care: a feasibility trial’, *American Journal of Hospice and Palliative Medicine*, *38*(12), 1488–1494. United Kingdom	To understand the feasibility of repeated personalized virtual reality sessions in a palliative care population	A feasibility randomized controlled trial	Personalized and non-personalized VR, using an intervention and a control group Described as an intervention
Ryu, S. and Price, S.K. (2021) ‘Embodied storytelling and meaning-making at the end of life: VoicingHan avatar life-review for palliative care in cancer patients’, *Arts & Health*, 1–15. United States of America	To present the qualitative analysis of the VoicingHan project based on the avatar videos which emerged as the artefacts of the feasibility study by Dang et al. (2021)	Retrospective qualitative analysis of avatar videos from an earlier feasibility study conducted by Dang et al. (2021)	An avatar-facilitated life review Described as an intervention

### VR description

Within the review, it was important to ascertain how the use of VR was described in the context of patient care. Across the 10 papers, most (*n* = 7) described VR as ‘an intervention’. Lloyd and Haraldsdottir^29^ described it as ‘a therapeutic intervention’. Ferguson et al.^22^ used the term ‘a therapeutic recreation’, while Nwosu et al.^30^ described it as ‘distraction therapy’ (Table 4). This was a vital observation as it gave context and some understanding of the possible motivations for using VR in PC settings, which mainly appears to be related to patient-centred support strategies.

**Table 4.** Patient profiles, palliative care settings, VR facilitators.

Patient profiles and palliative care settings	Virtual reality facilitators	Paper
People with terminal cancer at two PC wards	Medical staff	Niki et al.^27^
Patients diagnosed with life-limiting illness, resident at an inpatient freestanding hospice facility	Hospice social worker and a research team	Johnson et al.^26^
Hospitalized adults age 18 years and older, with a PC consult	PC inpatient team, music therapist	Brungardt et al.^23^
Hospice patients living with dementia cared for by a local hospice agency	Researcher	Ferguson et al.^22^
Advanced cancer patients, age range 18–70 years, assisted with a home PC programme	Psychologists and a physician	Moscato et al.^24^
Ambulatory oncology patients aged >18 years at a supportive care clinic at a National Cancer Institute	PC physician, PC nurse, clinic nurse, an art professor and research assistants	Dang et al.^25^
Adults with progressive life-limiting conditions under the care of a hospice (inpatient or outpatient)	Hospice clinical team (doctors, nurses and allied health professionals) and one researcher	Perna et al.^28^
Adults diagnosed with an advanced life-limiting condition, who are inpatients or were attending the outpatient unit and expected to be in the hospice for at least 1 week and with a life expectancy over 1 month and who have been in the hospice for at least 24 h	Hospice clinical staff, hospice researcher, research fellow×2, and a highly experienced VR facilitator	Lloyd and Haraldsdottir^29^
Cancer patients at a PC outpatient clinic	Artists, research assistants, PC physician and nurse	Ryu and Price^31^
Patients and caregivers in two specialist PC inpatient units	Clinical staff	Nwosu et al.^30^

#### Review question 1: What types of VR modalities have been used and reported in PC environments?

Within the review, VR experiences in PC were presented in varied ways, but fundamental to all was an interaction between patients and VR technology. Some presented as meditative/relaxing experiences, while others offered exciting experiences or travel experiences. Meditative and relaxing VR experiences involved either photorealistic still images or animated videos, some with and without music, and some offered guided meditation. Visually, options included natural beauties such as beaches, forests, waterfalls, parks, city landscapes and mountain landscapes. In other cases, patients had the option of an ‘exciting’ experience with choices involving simulations of roller coaster rides, rocket launches and space travel. Another option was travel experiences, which allowed patients to travel virtually to a choice of destinations, such as visiting somewhere they always wanted to go or revisiting a favourite destination from their past travels. Two papers reported on life review interventions combined with kinetic digital representations (i.e. avatars); in these cases, patients’ voices, gestures, and movements were synchronized onto an avatar in a virtual environment. All VR experiences used specialist technology involving hardware and software with online content which was either purchased or freely available (Table 5).

**Table 5.** VR modalities.

VR hardware	VR software	Reporting paper
VR headset HTC VIVE	Free VR software Google Earth VR	Niki et al.^27^
Samsung Gear VR technology	Photorealistic still images of popular real-world destinations and landscapes combined with an audio experience, animated videos, simulations of rocket launches and space travel (Apollo 11 and Hello Mars), simulation of a roller coaster (Coaster)	Johnson et al.^26^
Oculus Go VR headset (Facebook; Menlo Park, CA)	Nature-based videos from free online content (Atmosphaeres, Germany)	Brungardt et al.^23^
The Mirage Solo with Daydream Business Edition head-mounted VR headset and technology	YouTube VR 360 beach scene video	Ferguson et al.^22^
Mirage Solo VR (LENOVO S.r.l.)	Non-interactive contents consisted of immersive 360° videos with different natural and relaxing scenarios, such as a seascape, a park, a waterfall, the London Bridge, and a mountain landscape Interactive content consisted of a basic skill game called ‘Yuma's World’	Moscato et al.^24^
Perception Neuron MoCap system	VoicingHan – an avatar storytelling platform	Dang et al.^25^, Ryu and Price^31^
Google Daydream headset using Google Pixel XL.	Flix Films from publicly available video libraries such as YouTube	Perna et al.^28^
VR headsets, headphones with VR technology ‘room-scale’	Virtual destination chosen by user	Lloyd and Haraldsdottir^29^
Samsung Gear VR system	Oculus Gear VR store downloads including a 5-min guided relaxation video of a beach, a 10-min guided meditation through a computer-generated forest and a 5-min video roller coaster ride	Nwosu et al.^30^

#### Review question 2: What patient groups has VR been used with and in what context (e.g. inpatient care or home care), and who was involved in facilitating the VR experience?

All patients involved in VR experiences were adults receiving PC as either inpatients or outpatients. Some were inpatients in specialised hospice facilities, while others were inpatients on general hospital wards with hospice care consult. Some were attending PC clinics or under the care of home PC programmes. Broad terms were used to describe patients’ health status, for example, life-limiting illness, advanced life-limiting conditions, progressive life-limiting conditions, end-of-life with dementia, oncology, advanced cancer and terminal cancer. VR experiences were coordinated and facilitated by multidisciplinary professionals working with or connected to PC services. Most papers (*n* = 9) report the involvement of hospice-based professionals such as PC inpatient teams, PC physicians, PC nurses, hospice clinical staff teams, hospice social workers and medical staff. In addition, most papers (*n* = 6) mention the involvement of dedicated research staff such as hospice researchers, research assistants and research fellows. Allied health and other professionals included a psychologist, music therapist, art professor and artists. Of note, only one paper, Lloyd and Haraldsdottir,^29^ mentioned the involvement of a highly experienced VR facilitator. There were no other mentions of technology/VR specialists or technology specialism among those involved in facilitation (Table 4).

#### Review question 3: How have VR interventions been evaluated to date, i.e. what outcomes are measured?

All papers report on VR patient experiences in PC; however, the type of evaluations and outcomes measured are varied. A summary is provided in Table 6, which presents three categories of outcomes measured in the review papers: (a) patient outcomes, (b) user experience and (c) other stakeholder perspectives.

**Table 6.** Types of evaluations or patient outcomes measured.

Type of evaluation/outcome measure	Examples and reporting papers
Patient outcomes	Physical and psychological symptoms – Niki et al.^27^, Ferguson et al.^22^, Dang et al.^25^, Moscato et al.^24^ Functional assessment – Ferguson et al.^22^ Behavioural changes – Ferguson et al.^22^ Physiological signals associated with pain, anxiety and depression via a smart wristband (e.g. electro dermal activity, heart rate, skin) – Moscato et al.^24^ Quality of life measures – Dang et al.^25^, Moscato et al.^24^ Spiritual well-being – Dang et al.^25^
User experience	Numerical Rating Scale (NRS) for the assessments of participants’ level of fun, happiness pre- and post-VR experience – Niki et al.^27^ User perceptions of the intervention in relation to usability, likeability and perceived benefit – Johnson et al.^26^ User experience regarding feasibility, usability and acceptability using the System Usability Scale (SUS) – Brungardt et al.^23^ Participant views and perceptions of the VR experience (interviews) – Brungardt et al.^23^ Patients views and perceptions – Ferguson et al.^22^ Participant acceptability and tolerance of VR intervention – Ferguson et al.^22^ User data, amount of times used and usage time, preferences for interactive or non-interactive content – Moscato et al.^24^ Patient adherence, recruitment rate, acceptability and comfort of study procedures – Dang et al.^25^ The length of time required to set up equipment, time to set up the patient and the total session length of the avatar-facilitated life review – Dang et al.^25^ User experience of the VR session via observation and interviews – Lloyd and Haraldsdottir^29^ Retrospective qualitative analysis of patient experience via avatar videos – Ryu and Price^31^ Patient perceptions of VR use – Nwosu et al.^30^
Other stakeholder perspectives	Behavioural changes after VR experience measured by contacting the participant's primary caregiver – Ferguson et al.^22^ Caregiver’s perceptions of VR use – Nwosu et al.^30^ Staff perceptions regarding VR use and helpfulness in clinical practice – Nwosu et al.^30^ Public opinion views on how to support VR adoption in PC – Nwosu et al.^30^

##### Patient outcomes

Across the review, the impact of VR experiences on patient outcomes was measured in a variety of ways. Reported measurable outcome indicators included quality of life assessments, physical and psychological symptoms, functional assessments, physiological signals, spiritual well-being, and behavioural changes. A total of 11 different assessment tools, scales or instruments are reported (Table 7), and most were used as pre- and post-intervention measures. The most commonly reported assessment tool was the *Edmonton Symptom Assessment System* (ESAS), which is a validated quantitative instrument for self-reported symptom screening and monitoring for patients in various healthcare settings including PC.^32^

**Table 7.** Examples of assessment/measurement tools used and reporting papers.

Edmonton Symptom Assessment System (ESAS) – Dang et al.^25^, Johnson et al.^26^, Niki et al.^27^, Perna et al.^28^, Moscato et al.^24^ Numerical Rating Scale (NRS) for the assessments of dizziness and headache – Niki et al.^27^ The Functional Assessment Staging (FAST) Scale – Ferguson et al.^22^ The Pain Assessment in Advanced Dementia (PAINAD) Scale – Ferguson et al.^22^ Cancer-related symptomatology – Moscato et al.^24^ The Brief Pain Inventory (BPI) – Moscato et al.^24^ The Hospital Anxiety and Depression Scale (HADS) – Moscato et al.^24^ European Organization for Research and Treatment of Cancer Quality of Life Questionnaire Core 30 (EORTC QLQ-C30) – Dang et al.^25^ The Functional Assessment of Chronic Illness Therapy (FACIT) Spiritual Well-Being Scale (FACIT-Sp) – Dang et al.^25^ Health-related quality of life (HRQoL) – Dang et al.^25^ Assessment of Chronic Illness Therapy–Spiritual Well-Being Scale (FACIT-Sp) – Dang et al.^25^

##### User experience

Primary and/or secondary endpoints for most studies (*n* = 9) centred on the feasibility of implementing VR experiences in PC settings. Thus, user experiences were evaluated through patient interviews and observations and/or by using numerical scales. Outcome measures included subjective indicators, for example, patient-reported adherence, tolerance, acceptability and comfort of VR interventions,^22,25^ usability, likeability, preferences and perceived benefit,^25,26^ and finally perceived level of fun, happiness and enjoyment pre- and post-VR experiences.^27^

##### Other stakeholder perspectives

In two papers, VR use and patient outcomes were evaluated from stakeholder perspectives. Ferguson et al.^22^ monitored patient behavioural changes by contacting the participant's primary caregiver after the intervention. Nwosu et al.^30^ explored caregiver and staff perceptions regarding VR use in clinical practice and elicited public views/opinions on how to support VR adoption in PC at a public engagement event.

#### Review question 4: ‘What evidence base (if any) exists on the clinical effectiveness of VR in PC?’

The evidence on the clinical effectiveness of VR in PC is limited (Table 8). Primary and/or secondary endpoints for most papers (*n* = 9) centred on the feasibility of implementation and general VR user tolerability and acceptance. Qualitative and quantitative data on patient outcomes such as symptom relief or health benefits/risks is limited and originates from studies conducted in single geographical locations with small sample sizes. For example, in relation to quality of life measures, Dang et al.^25^ found no significant pre-test/post-test changes for health-related quality of life (HRQoL) scores. In relation to physical and psychological symptoms, patients reported perceived improvements in their symptoms related to underlying health conditions (e.g. improvements in pain, tiredness, body relaxation, breathing, depression, and anxiety). In addition, an increased sense of well-being and happiness was reported, as well as positive feelings of respite from the burden of being in the hospital.^23,27^ However, in the studies that used validated assessment tools, scales or instruments, there were minor or no improvements, with little statistical significance overall. Dang et al.^25^ report minor improvements or no change to the ESAS scores. Johnson et al.^26^ reported an overall trend of improvement in the ESAS scores of several symptoms after the VR intervention, but none were significantly significant. Perna et al*.*^28^ reported no overall statistical difference in pre- and post-ESAS scores between those who received the VR intervention and those in the control group. In contrast, one paper reported significant improvements in ESAS items related to pain, depression, anxiety, well-being and shortness of breath immediately after the VR experiences.^29^ Moscato et al.^24^ also used the *Hospital Anxiety and Depression Scale* (HADS) and the *Brief Pain Inventory* (BPI), but no significant improvements were reported here. Similarly, Ferguson et al.^22^ reported no significant change in the *Pain Assessment in Advanced Dementia* (PAINAD) scores. Within the review, only one paper measured physiological signals (i.e. electro dermal activity, heart rate, skin temperature and activity index) via a smart wristband; however, no statistically significant changes before, during and after the VR sessions were reported.^24^ One paper reported on spiritual well-being and reported that more patients showed improvements in their total scores on the *Assessment of Chronic Illness Therapy-Spiritual Well-Being Scale* (FACIT-Sp) than not, albeit overall pre-test/post-test changes were not significant.^25^ With regard to behavioural and psychological symptoms of dementia, Ferguson et al.^22^ reported no significant changes to baseline after VR experiences. However, an increase in pain scores for two patients and worsened behavioural and psychological symptoms of dementia at follow-up for two more patients was documented (one with increased crying and one with increased hallucinations). Four papers reported no negative effects or mild negative effects associated with VR use.^23,27,29,30^

**Table 8.** Evidence of clinical effectiveness relating to virtual reality use in palliative care.

Patient outcomes	**Quality of life measures** No significant pre-test/post-test changes for health-related quality of life (HRQoL) scores – Dang et al.^25^ **Physical and psychological symptoms** Significant improvements were observed for pain, tiredness, drowsiness, shortness of breath, depression, anxiety and well-being, as well as fun and happiness in pre- vs. post-VR travel score – Niki et al.^27^ No statistically significant changes; however, there was an overall trend of improvement in the ESAS-R scores of several symptoms after the VR intervention, namely, pain, tiredness, drowsiness, depression, and anxiety – Johnson et al.^26^ Statistically significant change in pre- and post-intervention Revised Edmonton Symptom Assessment (ESAS-R) scores for lack of appetite. However, the authors acknowledged that this result may be misleading due to difficulties with the instrument used – Johnson et al.^26^ Participants described physical changes, including improved pain, decreased chest tightness, body relaxation and positive changes in breathing – Brungardt et al.^23^ 10 participants described experiencing respite from their current situation of being hospitalized – Brungardt et al.^23^ Participants reflected feeling surprised by their emotions, including one participant who was tearful while debriefing with the music therapist – Brungardt et al.^23^ Majority of participants had no change in the Pain Assessment in Advanced Dementia (PAINAD) score – Ferguson et al.^22^ Anxiety, depression on the Hospital Anxiety and Depression Scale (HADS) and pain using the Brief Pain Inventory (BPI) did not change significantly between days 1 and 4. However, the Edmonton Symptom Assessment Scale (ESAS) items related to pain, depression, anxiety, well-being and shortness of breath collected immediately after the VR sessions showed a significant improvement – Moscato et al.^24^ Edmonton Symptom Assessment System (ESAS) scores for well-being showed improvements or no change in 9 out of 11 patients – Dang et al.^25^ Total Edmonton Symptom Assessment System (ESAS) scores improved for 6 out of 11 patients – Dang et al.^25^ The mean Edmonton Symptom Assessment System-Revised (ESAS-R) scores dropped following each session; however, this was not statistically significant. There was no overall statistical difference in the mean difference in pre and post ESAS-R scores overall between those who received the intervention and those in the control group – Perna et al.^31^ **Physiological signals** The physiological parameters via smart wristband (i.e. electro dermal activity, heart rate, skin temperature and activity index) showed no statistically significant changes before, during and after the VR sessions – Moscato et al.^24^ **Spiritual well-being** No significant pre-test/post-test changes for spiritual well-being. However, more patients showed improvements in their total scores on the Assessment of Chronic Illness Therapy–Spiritual Well-Being Scale (FACIT-Sp) than not – Dang et al.^25^ **Behavioural changes** Majority had no changes after VR experience to their baseline behavioural and psychological symptoms of dementia (BPSD) – Ferguson et al.^22^ **Negative outcomes** A few participants reported an increased Edmonton Symptom Assessment System (ESAS) scores after VR travel; however, none reported moderate/severe symptoms. No participants complained of serious side effects – Niki et al.^27^ None of the participants reported negative physical responses – Brungardt et al.^23^ VR was stopped early in two of the participants due to a 2-point increase in PAINAD score – Ferguson et al.^22^ Behavioural and psychological symptoms of dementia (BPSD) were reported to have worsened in two (8%) of the participants at follow-up (one with increased crying and one with increased hallucinations) – Ferguson et al.^22^ Negative responses were offered very infrequently and were mild – Lloyd and Haraldsdottir^29^ No major complication were noted – Nwosu et al.^30^
User experience	**Feasibility, usability and acceptability** Participants scored usability above average – Brungardt et al.^23^ There was no significant difference between dementia type and usage time or dementia severity and usage time – Ferguson et al.^22^ Acceptability by patients was high, and all agreed or strongly agreed they would participate in the avatar session again, would recommend it to others, and found the experience beneficial – Dang et al.^25^ It is feasible to complete repeated VR sessions within a PC population – Perna et al.^28^ It is feasible to use VR therapy in PC. Two participants reported minor problems (heaviness of the headset, difficulty in adjusting the head straps and problems focusing the image) – Nwosu et al.^30^ **Likeability, preferences, perceived benefit** Rated as moderately liked and moderately beneficial – Johnson et al.^26^ Participants described the intervention as comfortable and easy to engage in – Brungardt et al.^23^ 53% chose the highest rating in satisfaction – Brungardt et al.^23^ No significant association between type of dementia and enjoyment of VR, FAST score and enjoyment of VR, type of dementia and whether a participant would want to do VR again – Ferguson et al.^22^ Of the 25 participants, 14 (56%) reported enjoying VR and 12 (48%) would do it again – Ferguson et al.^22^ Most participants spoke favourably of VR–MT, describing pleasant emotional and physical responses – Brungardt et al.^23^ The majority of patients had strong, positive evaluations – Dang et al.^25^ Overall, participant responses were generally positive towards their experience of VR with some neutral responses given. VR sessions were acceptable for people within the hospice environment. The majority of participants enjoyed the experience. Many expressed joy and delight at the process – Lloyd and Haraldsdottir^29^ Most participants had a positive experience of the VR. All participants indicated that they would like to use the VR again – Nwosu et al.^30^
Other stakeholder perspectives	**Staff and public perspectives** Staff rated VR as helpful, recommended VR, and were willing to use VR in the future. Staff identified barriers to VR use – Nwosu et al.^30^

In addition to the subjective and objective patient outcomes reported in the findings of the review papers, most papers also discuss potential VR benefits and risks; however, these are projected possibilities rather than actual research findings. Possible benefits centre on improved physical health, well-being and quality of life for patients.^22–24,26–28^ In addition, improvements in psychological and emotional well-being are proposed.^23,26,27,29,31^ Dang et al.^25^ highlighted the possibility of improved spiritual well-being. Interestingly, Ryu and Price^31^ suggested that the multi-stakeholder involvement required for VR interventions contributes to cross-disciplinary and cross-cultural benefits for all involved. Conversely, many risks associated with VR use in PC were emphasized. Brungardt et al.^23^ and Dang et al.^25^ highlighted the emotional risks around unexpected or surprising emotions, resulting from VR interventions. Ferguson et al.^22^ reported physical health risks such as increased pain scores and/or worsening symptoms. Infection control risks were highlighted, particularly around the decontamination of reusable technological equipment.^22,23^ Technical issues were cited as possible complications or risks, for example, the risk of technical malfunctions and the need for ongoing device calibrations.^22,25,26,30^ In addition, the portability and heaviness of wearable equipment such as head-mounted devices needed consideration to avoid harm to patients.^22^ Timing as a risk consideration was a multifaceted phenomenon; the time needed for staff involved in designing, developing and implementing VR experiences was significant. Substantial time investment on behalf of patients was also a possible encumbrance. Brungardt et al.^23^ highlighted that the timing of VR interventions was important to minimize risk to patients and avoid adding unnecessary burdens.

#### Review question 5: What guidelines/policies exist regarding the use of VR in PC?

Across the included review papers, there were no reports of pre-existing policies, procedures, protocols or guidelines (PPPGs) utilised in relation to the design, development or implementation of VR experiences in PC. Similarly, there was no evidence in the findings regarding the new development of VR-related PPPGs. However, it is acknowledged that this review included primary research studies only, and it is likely that a broader search and inclusion of other sources of information such as grey literature would have highlighted VR-related PPPGs.

## Discussion

VR use in the context of healthcare is increasing, and although its utility in PC has been reported as promising,^26^ it is still developing in this context. The objective of this scoping review was to chart the literature on VR use in PC, identifying any evidence relating to biopsychosocial patient outcomes which could support its use in practice. Over a 10-year period from 2011 to 2021, we identified only 10 papers of 993 screened that specifically reported empirical research on VR use in adult PC. The paucity of primary research may reflect the recent emergence of VR use in this sector^2–4^; however, consideration must be given to the health status of the population which was described in these studies as having advanced cancer, life-limiting conditions, terminal illnesses, and at the end of life.

In the studies we reviewed, PC was provided in different healthcare settings including hospice and home care, and VR interventions were facilitated by multidisciplinary healthcare professionals. Of note, only one study^29^ mentioned the involvement of a VR specialist, which is of concern, given the evolving nature of VR and immersive technologies in healthcare. VR interventions need to be supported by multidisciplinary teams with specialism in technology, PC, psychology and other professionals/specialisms as appropriate to the nature of the VR intervention.

In all papers, VR use in PC was described as an intervention or a therapy, where the primary motivation for its use was complimentary psychosocial support for patients. These VR interventions were short-term recreational or relaxation experiences focused on benefiting quality of life,^26^ improving emotional well-being^23,31^ and creating cognitive distraction^22^ and/or as an adjuvant therapy to relieve psychological symptoms such as anxiety, depression and pain.^24^ The primary motivation for the VR intervention is important to consider as this will influence expectations of intervention outcomes and the involvement of appropriate professionals. For example, several studies referred to VR as a ‘therapeutic intervention’ suggesting that the intervention should be evidence-based and facilitated by an intervention specialist or therapist. According to Helmchem^33^ therapeutic effectiveness can only be assessed by relating the effects of a therapeutic intervention to a therapeutic aim. Given that PC aims to holistically and individually address the physical and psychological needs of patients with advanced, life-limiting illness,^34^ more robust longitudinal studies of effectiveness of therapeutic interventions using VR need to be conducted.

Several studies in our review utilised VR as an intervention for life review therapy and reminiscence. Traditional life review therapy requires specialised training and facilitation by a life review therapist, as memories and life events can be triggered during review and could result in negative emotional consequences.^25^ However, in this review there was no evidence that specialists in life review, psychotherapists or psychologists were involved in the reported studies. Although there was some evidence from patient self-reports of increased sense of emotional well-being and perceived improvements in physical condition, and in most studies, no major complications of VR interventions were reported, negative responses such as worsened behavioural and psychological symptoms and an increase in pain scores post-VR experiences were evident in research with persons with dementia.^22^ The suitability of VR interventions for specific cohorts in receipt of PC is in question. Although there were few negative responses to VR reported, the possibility of physical risks such as infection or motion sickness^23^ and unexpected emotions^25^ was mentioned. Unexpected emotional responses can result from immersion in a virtual world; therefore, there is a need to be cognisant of ethical responsibility and risk of participant burden in conducting research using VR. Madary and Metzinger^35^ in their publication of ethical concerns that may arise from research and personal use of VR recommended that, at the very least, researchers follow the principle of non-maleficence: do no harm. Bradley et al.^36^ argued however that although ethics must remain at the forefront of PC research, being overly reticent towards participant burden can reduce the valuable contributions made to healthcare interventions by research, which is only made possible by participant involvement. Therefore, a balance needs to be struck between the involvement and exclusion of participants on the grounds of vulnerability and should focus more on the enablement and protection measures in place within the research process.^37^

In our review, published evaluations of VR use in adult PC have provided favourable evidence of feasibility, acceptability and general positivity towards its use. While there is some evidence of perceived improvements in physical conditions, only one study^24^ reported on objective measures of physiological signals (electro dermal activity, heart rate and skin temperature) associated with pain, anxiety and depression via a smart wristband. This highlights the need for a more holistic research approach in future studies which incorporates a variety of outcome measures across biopsychosocial dimensions, an approach that supports the addition of objective physiological data collection, monitoring and protection of the person and delivers appropriate support as required during the research process.

Evidence for the clinical effectiveness of VR interventions or psychosocial benefit is limited due to small sample sizes and the use of mainly self-reported outcome measures. The most reported measure was the ESAS,^32^ and although minor improvements in ESAS scores were found, no statistically significant changes were recorded in symptoms pre- and post-VR interventions.^25,26,28^ Diverse assessment measures were employed to measure outcomes of VR interventions creating difficulty with comparison.

Since our review was conducted, new primary research studies have been published.^7–9,38^ They all identify as feasibility or pilot VR studies using mixed methods in PC contexts. All have small sample sizes (range 6–45 participants) and self-reported patient outcomes, which were measured using varied methods. Positive patient outcomes are reported such as reduced pain and pain-related symptoms,^7–9^ as well as improved ESAS scores.^38^ While these studies further corroborate the feasibility and acceptability of VR, they all advocate for larger randomized control trials to determine clinical effectiveness of VR use in PC. What remains uncertain on completion of this review is how patient outcomes correlate with VR clinical effectiveness, and how to measure such outcomes in larger randomized trials. There is a need for caution with comparison and synthesis of results in this area due to the diversity of VR applications used in PC and acknowledging the many varied approaches used to measure different patient outcomes across the biopsychosocial model of care. Similarly, Brickhead et al.^39^ advocate the need for consensus on how best to develop and evaluate VR treatments in a scientifically robust way. Furthermore, they recommend that randomized controlled studies which evaluate efficacy should only occur after sufficient research is conducted in the areas of VR content development, feasibility, acceptability, tolerability, and initial clinical efficacy. Future research designs should take cognisance of specialist methodological considerations and recommendations relating to technology-enhanced therapeutic interventions, which are still developing in the healthcare sector.

## Limitations

To strengthen this scoping review, structured systematic frameworks^15–19,40^ were adhered to, and a priori protocol was registered.^21^ However, by nature, scoping reviews are limited by their broad questions and searching methods, and it is possible that literature may have been missed in the protocol searching or study screening and searching phases. This review was also limited by the 10-year searching timeframe; however, this timeframe aligns with the resurgence of VR across various industries such as the entertainment, education and industry sectors, which means that from a technological and expertise perspective, VR is now more accessible and available than before and more likely to be used in healthcare contexts.^2–4^ The timeframe was also substantiated in this review as the final included papers were recent ranging the years 2019–2021. Another limitation was the inclusion of English language–only papers, this was linked to the authors’ time, language and resource constraints, and it is acknowledged that a search without language constraints would yield more results. A further limitation was the inclusion of primary research studies to the exclusion of other sources of information. While scoping methodologies can opt to leave the sources of information open to all types of evidence (e.g. grey literature),^18^ this review did impose limits here to capture sources of information that were more likely to report on patient outcomes and therefore align more closely with the research objective and primary question. Albeit, despite the ‘type of sources’ limitation, the inclusion of ‘any research design’ in the primary research studies did broaden the scope in capturing the diversities and pluralities of this topic.

## Conclusion

This scoping review charts the literature on VR use in PC, identifying evidence relating to biopsychosocial patient outcomes which could support its use in practice. This evidence is presented under subquestions which address pertinent clinical aspects of VR use in PC contexts. There is evidence that VR is being used with patients receiving PC in a variety of clinical contexts, and data around VR usability, feasibility and acceptability in patient care contexts appears positive; however, the evidence relating to patient outcomes ensuing from VR use is limited. VR is gathering momentum in PC and potentially is a helpful intervention, but more research is needed to underpin the evidence base supporting its application, particularly in understanding the impact on biopsychosocial patient outcomes and ascertaining the best approach for measuring intervention effectiveness.

## Implications of the findings for research

Our review found a paucity of well-designed large-scale studies that trialled specific well-defined VR interventions in this population. Future research on VR interventions in adult PC should focus on clinical effectiveness research using bio-physiological, psychological and behavioural indicators. As most of the previous research was conducted as pilot short-term investigations, future studies should be of longitudinal design but powered to adjust for attrition considering the vulnerability of the population. The potential barriers and facilitators to ‘real-world’ implementation of VR interventions could be explored in an effectiveness–implementation hybrid research design that would test the effects of VR as a clinical intervention while observing and gathering information on the implementation of the intervention, including any challenges associated.^41^

## Supplemental Material

sj-docx-1-dhj-10.1177_20552076231207574 - Supplemental material for Virtual reality use and patient outcomes in palliative care: A scoping review Click here for additional data file. Supplemental material, sj-docx-1-dhj-10.1177_20552076231207574 for Virtual reality use and patient outcomes in palliative care: A scoping review by Mairead Moloney, Owen Doody, Martina O’Reilly, Michael Lucey, Joanne Callinan, Chris Exton, Simon Colreavy, Frances O’Mahony, Pauline Meskell and Alice Coffey in DIGITAL HEALTH

sj-docx-2-dhj-10.1177_20552076231207574 - Supplemental material for Virtual reality use and patient outcomes in palliative care: A scoping review Click here for additional data file. Supplemental material, sj-docx-2-dhj-10.1177_20552076231207574 for Virtual reality use and patient outcomes in palliative care: A scoping review by Mairead Moloney, Owen Doody, Martina O’Reilly, Michael Lucey, Joanne Callinan, Chris Exton, Simon Colreavy, Frances O’Mahony, Pauline Meskell and Alice Coffey in DIGITAL HEALTH

sj-docx-3-dhj-10.1177_20552076231207574 - Supplemental material for Virtual reality use and patient outcomes in palliative care: A scoping review Click here for additional data file. Supplemental material, sj-docx-3-dhj-10.1177_20552076231207574 for Virtual reality use and patient outcomes in palliative care: A scoping review by Mairead Moloney, Owen Doody, Martina O’Reilly, Michael Lucey, Joanne Callinan, Chris Exton, Simon Colreavy, Frances O’Mahony, Pauline Meskell and Alice Coffey in DIGITAL HEALTH

## References

[1] Pillai AS Mathew PS . Impact of virtual reality in healthcare: a review. Virtual Augment Reality Mental Health Treat 2019: 17–31. DOI: 10.4018/978-1-5225-7168-1.

[2] Chirico A Lucidi F De Laurentiis M , et al. Virtual reality in health system: beyond entertainment. A mini-review on the efficacy of VR during cancer treatment. J Cell Physiol 2016; 231: 275–287. 26238976 10.1002/jcp.25117

[3] Snoswell AJ Snoswell CL . Immersive virtual reality in health care: systematic review of technology and disease states. JMIR Biomed Eng 2019; 4: e15025.

[4] O’Connor S . Virtual reality and avatars in health care. Clin Nurs Res 2019; 28: 523–528. 31064283 10.1177/1054773819845824

[5] Oxford VR . Our Projects - Mental Health Areas | Oxford VR. https://oxfordvr.org/projects/ (accessed 02 December 2021).

[6] Falconer CJ Rovira A King JA , et al. Embodying self-compassion within virtual reality and its effects on patients with depression. BJPsych Open 2016; 2: 74–80. 27703757 10.1192/bjpo.bp.115.002147 PMC4995586

[7] Austin PD Siddall PJ Lovell MR . Feasibility and acceptability of virtual reality for cancer pain in people receiving palliative care: A randomised cross-over study. Support Care Cancer 2022; 30: 3995–4005. 35064330 10.1007/s00520-022-06824-x PMC8782583

[8] Guenther M Görlich D Bernhardt F , et al. Virtual reality reduces pain in palliative care—a feasibility trial. BMC Palliat Care 2022; 21: 1–9. 36195865 10.1186/s12904-022-01058-4 PMC9533542

[9] Kelleher SA Fisher HM Winger JG , et al. Virtual reality for improving pain and pain-related symptoms in patients with advanced stage colorectal cancer: A pilot trial to test feasibility and acceptability. Palliative and Supportive Care 2022; 20: 471–481. 35078545 10.1017/S1478951521002017 PMC9314453

[10] Repetto C Riva G . From virtual reality to interreality in the treatment of anxiety disorders. Neuropsychiatry 2011; 1: 31–43.

[11] World Health Organisation. Planning and implementing palliative care services: A guide for programme managers. Geneva, 2016.

[12] Martin JL Saredakis D Hutchinson AD , et al. Virtual reality in palliative care: A systematic review. Healthcare 2022; 10: 1222. MDPI. 35885749 10.3390/healthcare10071222 PMC9319274

[13] Mo J Vickerstaff V Minton O , et al. How effective is virtual reality technology in palliative care? A systematic review and meta-analysis. Palliat Med 2022;36: 1047–1058. 35635018 10.1177/02692163221099584 PMC9248003

[14] Carmont H McIlfatrick S . Using virtual reality in palliative care: A systematic integrative review. Int J Palliat Nurs 2022; 28: 132–144. 35452268 10.12968/ijpn.2022.28.3.132

[15] Arksey H O'Malley L . Scoping studies: towards a methodological framework. Int J Soc Res Methodol 2005; 8: 19–32.

[16] Levac D Colquhoun H O'Brien KK . Scoping studies: advancing the methodology. Implement Sci 2010; 5: 1–9. 20854677 10.1186/1748-5908-5-69 PMC2954944

[17] Peters MD Godfrey CM Khalil H , et al. Guidance for conducting systematic scoping reviews. JBI Evid Implement 2015; 13: 141–146. 10.1097/XEB.0000000000000050 26134548

[18] Peters MD Marnie C Tricco AC , et al. Updated methodological guidance for the conduct of scoping reviews. JBI Evid Synth 2020; 18: 2119–2126. 33038124 10.11124/JBIES-20-00167

[19] Pollock D Davies EL Peters MD , et al. Undertaking a scoping review: A practical guide for nursing and midwifery students, clinicians, researchers, and academics. J Adv Nurs 2021; 77: 2102–2113. 33543511 10.1111/jan.14743 PMC8049063

[20] Page MJ Mckenzie JE Bossuyt PM , et al. The PRISMA 2020 statement: an updated guideline for reporting systematic reviews. Int Surg J 2021; 88: 105906. 10.1016/j.ijsu.2021.105906 33789826

[21] Coffey A O'Reilly M Lucey M , et al. A Scoping review of evidence on use and effectiveness of virtual reality in palliative care: protocol. 2021. Epub ahead of print 21 December 2021. DOI: 10.17605/OSF.IO/9QCXW.

[22] Ferguson C Shade MY Blaskewicz Boron J , et al. Virtual reality for therapeutic recreation in dementia hospice care: A feasibility study. Am J Hosp Palliat Med 2020; 37: 809–815. 10.1177/1049909120901525 31975609

[23] Brungardt A Wibben A Tompkins AF , et al. Virtual reality-based music therapy in palliative care: A pilot implementation trial. J Palliat Med 2021; 24: 736–742. 33227225 10.1089/jpm.2020.0403 PMC8064967

[24] Moscato S Sichi V Giannelli A , et al. Virtual reality in home palliative care: brief report on the effect on cancer-related symptomatology. Front Psychol 2021; 12: 709154. 34630217 10.3389/fpsyg.2021.709154 PMC8497744

[25] Dang M Noreika D Ryu S , et al. Feasibility of delivering an avatar-facilitated life review intervention for patients with cancer. J Palliat Med 2021; 24: 520–526. 32896200 10.1089/jpm.2020.0020

[26] Johnson T Bauler L Vos D , et al. Virtual reality use for symptom management in palliative care: a pilot study to assess user perceptions. J Palliat Med 2020; 23: 1233–1238. 31895637 10.1089/jpm.2019.0411

[27] Niki K Okamoto Y Maeda I , et al. A novel palliative care approach using virtual reality for improving various symptoms of terminal cancer patients: a preliminary prospective, multicenter study. J Palliat Med 2019; 22: 702–707. 30676847 10.1089/jpm.2018.0527

[28] Perna L Lund S White N , et al. The potential of personalized virtual reality in palliative care: A feasibility trial. Am J Hosp Palliat Med 2021; 38: 1488–1494. 10.1177/1049909121994299 PMC8641032 33583203

[29] Lloyd A Haraldsdottir E . Virtual reality in hospice: improved patient well-being. BMJ Support Palliat Care 2021; 11: 344–350. 10.1136/bmjspcare-2021-003173 34215568

[30] Nwosu AC Mills M Roughneen S , et al. Virtual reality in specialist palliative care: A feasibility study to enable clinical practice adoption. BMJ Support Palliat Care 2021: bmjspcare-2020-002327. DOI: 10.1136/bmjspcare-2020-002327. PMC10894832 33597168

[31] Ryu S Price SK . Embodied storytelling and meaning-making at the end of life: voicingHan avatar life-review for palliative care in cancer patients. Arts Health 2022; 14: 326–340. 34160335 10.1080/17533015.2021.1942939

[32] Hui D Bruera E . The Edmonton Symptom Assessment System 25 years later: past, present, and future developments. J Pain Symptom Manage 2017; 53: 630–643. 28042071 10.1016/j.jpainsymman.2016.10.370 PMC5337174

[33] Helmchem H . Assessment and application of therapeutic effectiveness, ethical implications of. In: Smelser NJ Baltes PB (eds) International encyclopedia of the social & behavioral sciences. Pergamon, 2001, pp.829–833, ISBN 9780080430768, DOI: 10.1016/B0-08-043076-7/00180-7.

[34] World Health Organization. WHO Definition of Palliative Care 2017, https://www.who.int/cancer/palliative/definition/en/ (accessed 02 December 2021).

[35] Madary M Metzinger TK . Real virtuality: A code of ethical conduct. Recommendations for good scientific practice and the consumers of VR-technology. Front Robot AI 2016; 3: 3.

[36] Bradley N Lloyd-Williams M Dowrick C . Effectiveness of palliative care interventions offering social support to people with life-limiting illness—a systematic review. Eur J Cancer Care (Engl) 2018; 27: e12837. Epub 2018 Mar 24. PMID: 29573500; PMCID: PMC6001732. 10.1111/ecc.12837 PMC6001732 29573500

[37] Doody O . Ethical challenges in intellectual disability research. Mathews J Nurs Health Care 2018; 1: 1–1.

[38] Seiler A Schettle M Amann M , et al. Virtual reality therapy in palliative care: A case series. J Palliat Care 2022: 08258597221086767. DOI: 10.1177/08258597221086767. 35293818

[39] Birckhead B Khalil C Liu X , et al. Recommendations for methodology of virtual reality clinical trials in health care by an international working group: iterative study. JMIR Ment Health 2019; 6: e11973. 10.2196/11973 PMC6374734 30702436

[40] McGowan J Sampson M Salzwedel DM , et al. PRESS peer review of electronic search strategies: 2015 guideline statement. J Clin Epidemiol 2016; 75: 40–46. Niki K, Okamoto Y, Maeda I, et al. A novel palliative care approach using virtual reality for improving various symptoms of terminal cancer patients: A preliminary prospective, multicenter study. J Palliat Med 2019; 22: 702–707. 10.1089/jpm.2018.0527. 27005575

[41] Curran GM Bauer M Mittman B , et al. Effectiveness-implementation hybrid designs: combining elements of clinical effectiveness and implementation research to enhance public health impact. Med Care 2012; 50: 217–226. PMID: 22310560; PMCID: PMC3731143. 22310560 10.1097/MLR.0b013e3182408812 PMC3731143

