# Supplementary material for: Virtual reality use and patient outcomes in palliative care: A scoping review
Source: Digit Health. 2023 Nov 1;9:20552076231207574. doi: 10.1177/20552076231207574 (PMC10621306; doi:10.1177/20552076231207574)
Supplement: sj-docx-1-dhj-10.1177_20552076231207574 - Supplemental material for Virtual reality use and patient outcomes in palliative care: A scoping review [file sj-docx-1-dhj-10.1177_20552076231207574.docx]

**Supplementary File 1 – Data Charting Table**

**Primary research question**

What knowledge and evidence exists about VR use and patient outcomes in palliative care?

**Research sub questions 1-5**

1. What types of VR modalities have been used and reported in palliative care environments?
2. What patient groups has VR been used with, and in what context (e.g., inpatient care or home care), and who was involved in facilitating the VR experience?
3. How have VR interventions been evaluated to date - i.e., what patient outcomes are measured?
4. ‘What evidence base (if any) exists on the clinical effectiveness of VR in palliative care?’**?**
5. What guidelines/policies exist regarding the use of VR in palliative care?

**Data Charting Table**

| **Author(s), Year of Publication, Origin** | **Aim/Purpose** | **Methods/ Methodology** | **Virtual Reality Description** | **Key findings relating to review questions** | | | | | |
| --- | --- | --- | --- | --- | --- | --- | --- | --- | --- |
|  |  |  |  | **Q1** | **Q2** | **Q3** | **Q4** | **Q5** | **Possible benefits and risks** |
| Brungardt, A., Wibben, A., Tompkins, A.F., Shanbhag, P., Coats, H., LaGasse, A.B., Boeldt, D., Youngwerth, J., Kutner, J.S. and Lum, H.D. (2021) ‘Virtual reality-based music therapy in palliative care: a pilot implementation trial’, *Journal of Palliative Medicine*, *24*(5), 736-742.  United States of America | To evaluate implementation measures of feasibility, usability, and acceptability of a VR-based music therapy (MT) intervention. | A pilot implementation study of a two-day VR–MT intervention using mixed methods.  Data collection: Patient data and participant interviews.  Data analysis: Descriptive statistics to summarize quantitative data using SAS, version 9.4.  Qualitative descriptive approach to analyze interviews. | VR experience with sound and music.  Described as an intervention. | Participant customized soundtrack with a music therapist for listening during a VR experience.  Video options included four nature-based videos from free online content (Atmo- sphaeres, Germany), downloaded onto the VR headset.  Oculus Go VR (Facebook; Menlo Park, CA) headset was used. | Hospitalized adults age 18 years and older (n=23), with a palliative care consult, estimated length of stay of at least two days, and diagnosis of cancer, heart failure, or end-stage renal disease.  VR facilitated by palliative care inpatient team, including a music therapist. | Patient data including reasons for enrolling/ declining/discontinuing.  Mortality data such as location of death.  User experience regarding feasibility, usability and acceptability using the System Usability Scale (SUS).  Participant views and perceptions of the experience via interviews. | Participants scored usability above average.  53% chose the highest rating in satisfaction.  Participants described the intervention as comfortable and easy to engage in.  Most participants spoke favourably of VR–MT, describing pleasant emotional and physical responses.  Participants described physical changes, including improved pain, decreased chest tightness, body relaxation, and positive changes in breathing.  None of the participants reported negative physical responses.  Participants reflected feeling surprised by their emotions, including one participant who was tearful while debriefing with the music therapist.  10 participants described experiencing respite from their current situation of being hospitalized. | Not identified in findings. | Physical health and wellbeing benefits.  Psychological and emotional benefits.  Emotional risks around unexpected or surprising emotions as a result of the intervention.  Infection control risks regarding the need for VR equipment decontamination between patient uses.  Timing of the intervention is important to minimise risk to the patient (consider times when more or less emotional / more or less symptoms / burden of care). |
| Dang, M., Noreika, D., Ryu, S., Sima, A., Ashton, H., Ondris, B., Coley, F., Nestler, J. and Fabbro, E.D., (2021) ‘Feasibility of delivering an avatar-facilitated life review intervention for patients with cancer’, *Journal of Palliative Medicine*, *24*(4), 520-526.  United States of America. | To determine the feasibility of an avatar-based intervention for facilitating life review in patients with advanced cancer. | Prospective trial to establish feasibility and acceptability of an avatar-based intervention for facilitating life review in patients with advanced cancer.  Data collection: Baseline questionnaires, avatar sessions, post-session surveys, and one-month follow-up questionnaires.  Data analysis methods not explicit. | VoicingHan - an avatar-facilitated life review.  Described as an intervention. | Life review intervention combined with a kinetic, digital representation (avatar) chosen by the patient (ranging 20-40 minutes intervention).  VoicingHan includes 5 environments (beach, mountain, city, living room, and empty space) and 64 customized avatar options, including 4 male and 4 female avatars at 4 developmental stages: child, teenager, adult, and elder.  Perception Neuron MoCap system was used to synchronize voice, gestures, and movements onto an avatar in a virtual environment | Ambulatory oncology patients age > 18 years (n=12) at a supportive care clinic at a National Cancer Institute.  VR facilitated by a palliative care physician, palliative care nurse, clinic nurse, an art professor and research assistants. | The primary outcomes assessed the feasibility of delivering an avatar-facilitated life review intervention as determined by patient adherence, recruitment rate, acceptability and comfort of study procedures.  Pre-intervention assessments included the ESAS, *European Organization for Research and Treatment of Cancer- Quality of Life Questionnaire Core 30* (EORTC QLQ-C30), and the *Functional Assessment of Chronic Illness Therapy (FACIT) Spiritual Well-Being Scale (FACIT-Sp).*  Patients’ perceived benefits using baseline questionnaires, avatar sessions, post-session surveys, and one-month follow-up questionnaire e.g. *health related quality of life* (HRQoL), *Assessment of Chronic Illness Therapy-Spiritual Well-Being Scale* (FACIT-Sp) and the *Edmonton Symptom Assessment System* (ESAS).  Secondary outcome measures included the length of time required to set up equipment, time to set up the patient, and the total session length of the avatar-facilitated life review. | No significant pre-test/post-test changes for spiritual wellbeing or *health related quality of life* (HRQoL) scores. However, more patients showed improvements in their total scores on the *Assessment of Chronic Illness Therapy-Spiritual Well-Being Scale* (FACIT-Sp) than not.  *Edmonton Symptom Assessment System* (ESAS) scores for well-being showed improvements or no change in 9 out of 11 patients.  Total *Edmonton Symptom Assessment System* (ESAS) scores improved for 6 out of 11 patients.  Changes in global health, functional scale, and physical symptom scale varied.  The majority of patients had strong, positive evaluations.  Acceptability by patients was high, and all agreed or strongly agreed they would participate in the avatar session again, would recommend it to others, and found the experience beneficial. | Not identified in findings. | Improved spiritual wellbeing.  Improved physical wellbeing, HRQoL, symptom burden and wellbeing.  Unexpected emotions and thinking of issues that one didn’t want to face/think about.  Minor technical malfunctions.  Length of time required to set up equipment, time to set up the patient, and the total session length of the avatar-facilitated life review.  Patient  setup time included a brief avatar tutorial demonstrating the  range of movements and lip-syncing capabilities, strapping  partial- or full-body connection to patients, and device calibration. |
| Ferguson, C., Shade, M.Y., Blaskewicz Boron, J., Lyden, E. and Manley, N.A. (2020) ‘Virtual reality for therapeutic recreation in dementia hospice care: A feasibility study’, *American Journal of Hospice and Palliative Medicine*, *37*(10), 809-815.  United States of America. | To explore acceptability, tolerability, and subjective experience of virtual reality (VR) as therapeutic recreation for hospice patients living with dementia. | A feasibility study using descriptive design.  Data collection: demographics from medical records. Pain survey tools/scales and semi-structured patient interview. Follow up with carers after the intervention about patient behavioural changes after VR experience using an investigator-derived form.  Data analysis: descriptive statistics using SAS version 9.4 and content analysis. | Virtual reality experience.  Described as a therapeutic recreation. | Participants viewed a beach scene using VR headset for approximately 30 minutes.  The experience involved a short YouTube VR 360 beach scene video - a 3.5-minute video looped for up to 12 times.  The Mirage Solo with Daydream Business Edition head mounted VR headset and technology were used.  VR facilitators not identified. | Hospice patients living with dementia (n=25) cared for by a local hospice agency.  VR facilitated by the researcher. | Participant acceptability and tolerance of VR intervention.  Demographic information from medical records and data on dementia type and severity using the *Functional Assessment Staging* (FAST) Scale.  The *Pain Assessment in Advanced Dementia* (PAINAD) Scale were assessed at baseline, every 5 minutes of headset use, and 5 minutes after headset removal for a total of up to 8 scores.  Perceived experience of VR use was measured using semi-structured interviews.  Behavioral changes after VR experience were documented on an investigator-derived form and were measured by contacting the participant’s primary caregiver > 3 hours after the intervention. | There was no significant difference between dementia type and usage time or dementia severity and usage time.  No significant association between  (1) type of dementia and enjoyment of VR, (2)  FAST score and enjoyment of VR,  (3) type of dementia and whether a participant would want to do VR again (4) FAST score and whether the participant would want to do VR again.  Majority of participants had no change in the *Pain Assessment in Advanced Dementia* (PAINAD) score. However, the VR was stopped early in 2 of the participants due to a 2-point increase in PAINAD score.  Majority had no changes after VR experience to their baseline behavioural and psychological symptoms of dementia (BPSD). However, BPSD were reported to have worsened in 2 (8%) of the participants at follow-up (1 with increased crying and 1 with increased hallucinations)  Of the 25 participants, 14 (56%) reported enjoying VR and 12 (48%) would do it again. | Not identified in the findings. | Enjoyment and enhanced quality of life.  VR scene may be understimulating or uninteresting to some.  Increased PAINAD scores.  Worsened BPSD.  Technical issues, cleaning of VR equipment, portability and heaviness of equipment need consideration. |
| Johnson, T., Bauler, L., Vos, D., Hifko, A., Garg, P., Ahmed, M. and Raphelson, M. (2020) ‘Virtual reality use for symptom management in palliative care: a pilot study to assess user perceptions’, *Journal of Palliative Medicine*, *23*(9), 1233-1238.  United States of America | To examine the utility of VR for palliative care patients. | A prospective single center, single-arm study.  Data collection: 10-question survey with two quantitative items and eight qualitative items.  Data analysis: Power analysis using SAS v9.4 | A virtual reality experience.  Described as an Intervention | All VR experiences included auditory and visual components, ranging from photorealistic still images to animated videos (one time, 30 minute duration).  VR options included - Still pictures of popular real-world destinations and landscapes.  Still pictures of virtual landscapes combined with an audio experience that guides the user through meditative exercises.  Simulations of rocket launches and space travel. (Apollo 11 and Hello Mars).  Simulation of a roller coaster (Coaster).  Samsung Gear VR technology was used. | Patients diagnosed with life limiting illness (n=12) resident at an inpatient free-standing hospice facility.  VR facilitated by hospice social worker and a research team. | User perceptions of the intervention in relation to usability, likeability and perceived benefit.  Symptom burden using the *Revised Edmonton Symptom Assessment scale* pre and post intervention | Positive and negative user perceptions of the VR experience. It was rated as moderately liked and moderately beneficial.  Statistically significant change in pre- and post-intervention *Revised Edmonton Symptom Assessment* (ESAS-r) scores for lack of appetite. However, the authors acknowledged that this result may be misleading due to difficulties with the instrument used.  No other statistically significant changes, however there was an overall trend of improvement in the ESAS-r scores of several symptoms after the VR intervention, namely pain, tiredness, drowsiness, depression, and anxiety. | Not identified in the findings. | An overall trend of improvement in the ESAS-r scores of several symptoms (although not statistically significant).  Positive emotional responses.  Training sessions needed before the intervention (approximately five minutes) on the use of the HMD and handheld controller.  Difficulties with VR operation.  Sore shoulders attributed to repeated adjustments of the head mounted device. |
| Lloyd, A. and Haraldsdottir, E. (2021) ‘Virtual reality in hospice: improved patient well-being’, BMJ Support Palliative Care, 11(3), 344-350.  Scotland | To trial VR technology and consider what benefits may emerge for hospice inpatients. | Qualitative exploratory study.  Data collection: observation and semi-structured interviews.  Data analysis: thematic analysis using NVivo12. | Virtual reality session.  Described as a therapeutic intervention. | One-off 30 minute VR session where participants were immersed in a virtual world, using ‘room-scale’ VR technology that tracks the user’s movement and relates them to the virtual world, the user choose a destination of choice, all guided by a highly experienced VR facilitator. | Adults diagnosed with an advanced life-limiting condition (n=19) who are inpatients or were attending the outpatient unit and expected to be in the hospice for at least 1 week and with a life expectancy over 1 month and who have been in the hospice for at least 24 hours.  VR facilitated by hospice clinical staff, hospice researcher, research fellow x 2, and a highly experienced VR facilitator. | User experience of the VR session via observation and interviews. | Overall, participant responses were generally positive towards their experience of VR with some neutral responses given.  Negative responses were offered very infrequently and were mild.  VR sessions were acceptable for people within the hospice environment.  The majority of participants enjoyed the experience.  Many expressed joy and delight at the process. | Not identified in the findings. | Enjoyable experience.  Positive impact on well-being for patients receiving palliative care through the capacity to transcend their current reality and to connect with another meaningful reality. |
| Moscato, S., Sichi, V., Giannelli, A., Palumbo, P., Ostan, R., Varani, S., Pannuti, R. and Chiari, L. (2021) ‘Virtual Reality in Home Palliative Care: Brief Report on the Effect on Cancer-Related Symptomatology’, *Frontiers in Psychology*, 12 (709154).  Italy | To assess the effect of an immersive VR-based intervention conducted at home on anxiety, depression, and pain over 4 days and to evaluate the short-term effect of VR sessions on cancer-related symptomatology. | A mixed methods pre-post single-arm study.  Data collection: Socio-demographic and clinical data from medical records. Questionnaire, survey scales and inventory. Wristband for physiological signals.  Data analysis: Statistical analyses. Physiological signals were performed in Matlab (Matlab, 2020). | Virtual reality-based intervention.  Described as an Intervention. | Four days of an immersive VR-based intervention conducted at home.  It was suggested to use VR in moments of psychophysical discomfort (e.g., pain and growing anxiety).  No set minimum or a maximum usage time or number of sessions.  Non-interactive contents consisted of immersive 360° videos with different natural and relaxing scenarios, such as a seascape, a park, a waterfall, the London Bridge, and a mountain landscape.  Interactive content consisted of a basic skill game called “Yuma’s World”  Mirage Solo VR (LENOVO S.r.l.) was used. | Advanced cancer patients (n=14), age range 18-70 years, assisted with a home palliative care programme.  VR facilitated by psychologists and a physician. | Socio-Demographic and Clinical Data.  User data e.g. amount of times used and usage time, preferences for interactive or non-interactive content.  Cancer-related symptomatology on day 1 and 4.  Anxiety and depression were measured by the *Hospital Anxiety and Depression Scale* (HADS)  Pain was measured using *the Brief Pain Inventory* (BPI).  Before and after each VR session, symptoms were collected by the *Edmonton Symptom Assessment Scale* (ESAS).  Participants wore a smart wristband measuring physiological signals (electro dermal activity, heart rate, skin temperature) associated with pain, anxiety, and depression to evaluate the psychophysiological effects of the VR. | Anxiety, depression on the *Hospital Anxiety and Depression Scale* (HADS), and pain using the *Brief Pain Inventory* (BPI) did not change significantly between days one and four.  However, the *Edmonton Symptom Assessment Scale* (ESAS) items related to pain, depression, anxiety, well-being, and shortness of breath collected immediately after the VR sessions showed a significant improvement.  The physiological parameters (electrodermal activity, heart rate, skin temperature, activity index) showed no significant changes before, during, and after the VR sessions.  A progressive reduction in electrodermal activity observed comparing the recordings before, during, and after the VR sessions, although these changes were not statistically significant. | Not identified in the findings. | Significant improvement in the *Edmonton Symptom Assessment Scale* (ESAS) items related to pain, depression, anxiety, well-being, and shortness of breath collected immediately after the VR sessions. |
| Niki, K., Okamoto, Y., Maeda, I., Mori, I., Ishii, R., Matsuda, Y., Takagi, T. and Uejima, E. (2019) ‘A novel palliative care approach using virtual reality for improving various symptoms of terminal cancer patients: a preliminary prospective, multicenter study’, *Journal of Palliative Medicine*, *22*(6), 702-707.  Japan | To verify whether simulated travel using virtual reality (VR travel) is efficacious in improving symptoms in terminal cancer patients. | Prospective, multicenter, single-arm study.  Data collection: medical records review, assessment tools and scales.  Data analysis: Statistical analyses. | Simulated travel using virtual reality.  Described as an intervention. | 30 minute VR travel session  VR headset HTC VIVE and the free VR software Google Earth VR. | People with terminal cancer (n=20) at two palliative care wards.  VR facilitated by medical staff. | Primary endpoint was the change in *Edmonton Symptom Assessment (ESAS)* score for each symptom before and after VR travel.  Secondary endpoints were the correlations between the experience types (participants who went to memorable places or participants who went to a place they had wanted to go to but never visited) and the effects on ESAS symptom scores.  Numerical Rating Scale (NRS) for the assessments of dizziness and headache,  Numerical Rating Scale (NRS) for the assessments of participants’ level of fun, happiness, and pre-VR travel expectations/post-VR travel satisfaction shortly before and immediately after VR travel. | Significant improvements were observed for pain, tiredness, drowsiness, shortness of breath, depression, anxiety and well-being, as well as fun and happiness in pre- vs. post-VR travel score.  A few participants reported increased *Edmonton Symptom Assessment (ESAS)* scores after VR travel, however none reported moderate/severe symptoms.  No participants complained of serious side effects. | Not identified in the findings. | Improvements in the *Edmonton Symptom Assessment Scale* (ESAS) for pain, tiredness, drowsiness, shortness of breath, depression, anxiety and well-being, as well as fun and happiness. |
| Nwosu, A.C., Mills, M., Roughneen, S., Stanley, S., Chapman, L. and Mason, S.R. (2021) ‘Virtual reality in specialist palliative care: a feasibility study to enable clinical practice adoption’ *BMJ Supportive & Palliative Care, 0, 1-5.*  United Kingdom | To (1) explore the feasibility of implementing VR therapy, for patients and caregivers, in a hospital specialist inpatient palliative care unit and a hospice, and (2) to identify questions for organisations, to support VR adoption in palliative care. | Quality improvement project.  Data Collection: Staff, patient and caregiver feedback and questionnaires.  A public engagement event (i.e. a modified world café method) to identify questions to support implementation of VR in palliative care settings.  Data analysis: Not stated. | Virtual reality distraction therapy.  Described as a distraction therapy. | VR experiences included a 5-minute guided relaxation video of a beach, a 10-minute guided meditation through a computer-generated forest and a 5-minute video rollercoaster ride.  Samsung Gear VR system used with experiences downloaded from Oculus Gear VR store. | Patients (n=12) and caregivers (n=3) in two specialist palliative care inpatient units.  Staff involved in the project (n=7).  People (lay representatives) at a public engagement event (n=6).  VR was facilitated by clinical staff. | Patient and caregivers perceptions of VR use.  Staff perceptions regarding VR use and helpfulness in clinical practice.  Public opinion views on how to support VR adoption in palliative care. | It is feasible to use VR therapy in palliative care.  Most participants had a positive experience of the VR.  All participants indicated that they would like to use the VR again.  No major complication were noted.  Two participants reported minor problems (heaviness of the headset, difficulty in adjusting the head straps and problems focusing the image).  Staff rated VR as helpful, recommended VR, and were willing to use VR in the future. Staff identified barriers to VR use as infection-control issues, issues with staff unfamiliar of how to use the equipment, and technical issues of ensuring the equipment was updated, charged and ready for use.  The public engagement event identified the following questions which need to be answered to support VR in palliative care: the purpose of VR;  intended population; supporting evidence; session duration; equipment choice; infection-control  issues;  content choice; setting of VR; person(s) responsible  for delivery and the maintenance plan. | Not identified in the findings. | Positive user experience.  Disorientation  noted by some participants.  Technical issues relating to setting up and charging the device.  Minor problems with heaviness and usability of the VR equipment. |
| Perna, L., Lund, S., White, N. and Minton, O. (2021) ‘The Potential of Personalized Virtual Reality in Palliative Care: A Feasibility Trial’, *American Journal of Hospice and Palliative Medicine*, *38*(12), 1488-1494.  United Kingdom | To understand the feasibility of repeated personalized virtual reality sessions in a palliative care population. | A feasibility randomized control trial.  Data collection: assessment tools and demographic information.  Data analysis: Statistical analyses using STATA v14.0. | Personalized and non- personalized virtual reality, using an intervention and a control group.  Described as an intervention. | 4-minute VR sessions once a week x 4 weeks.  Google Daydream headset using Google Pixel XL. The content was sourced and made possible for use in a clinical setting by Flix Films from publicly available video libraries such as YouTube. | Adults (n=26) with progressive life limiting conditions under the care of a hospice (at hospital or at home).  All VR interventions took place in the hospital (inpatient or outpatient settings).  VR facilitated by the hospice clinical team (doctors, nurses, and allied health professionals) and one researcher. | *Edmonton Symptom Assessment System-Revised* (ESAS-R) scores pre and post intervention. | It is feasible to complete repeated virtual reality sessions within a palliative care population.  The mean *Edmonton Symptom Assessment System-Revised* (ESAS-R) scores dropped following each session, however this was not statistically significant.  There was no overall statistical difference in the mean difference in pre- and post-ESAS-R scores overall between those who received the intervention and those in the control group. | Not identified in the findings. | Improvements in *Edmonton Symptom Assessment System-Revised* (ESAS-R) scores, however not statistically significant.  Time it takes to recruit suitable participants and implement intervention is significant. |
| Ryu, S. and Price, S.K. (2021) ‘Embodied storytelling and meaning-making at the end of life: VoicingHan avatar life-review for palliative care in cancer patients’, *Arts & Health*, 1-15.  United States of America. | To present the qualitative analysis of the VoicingHan project based on the avatar videos which emerged as the artifacts of the feasibility study by Dang et al (2021). | Retrospective qualitative analysis of avatar videos from an earlier feasibility study conducted by Dang et al (2021).  Observational analysis of the videos by unitizing the performance narratives into short segments and coding them using the template structure. | VoicingHan - an avatar-facilitated life review.  Described as an intervention. | Life review intervention combined with a kinetic, digital representation (avatar) chosen by the patient (ranging 20-40 minutes intervention).  VoicingHan includes 5 environments (beach, mountain, city, living room, and empty space) and 64 customized avatar options, including 4 male and 4 female avatars at 4 developmental stages: child, teenager, adult, and elder.  Perception Neuron MoCap system was used to synchronize voice, gestures, and movements onto an avatar in a virtual environment | Cancer patients (n=12) at a palliative care outpatient clinic.  Facilitated by a team including artists, research assistants, palliative care physician and nurse. | Retrospective qualitative analysis of the VoicingHan avatar videos to assess the construction and meaning-making made evident in the emotions and behaviors of the cancer patients during VoicingHan life review. | Physical and virtual bodies can and do work in concert to add depth, dimension and meaning into life, even in its most complex and challenging moments encountering the proximity of death. | Not identified in the findings. | VR based life reviews can add depth, dimension and meaning into life.  When VR interventions are created and implemented by professionals from diverse disciplines there are cross-disciplinary and cross-cultural benefits. |
