# Supplementary material for: Virtual reality use and patient outcomes in palliative care: A scoping review
Source: Digit Health. 2023 Nov 1;9:20552076231207574. doi: 10.1177/20552076231207574 (PMC10621306; doi:10.1177/20552076231207574)
Supplement: sj-docx-2-dhj-10.1177_20552076231207574 - Supplemental material for Virtual reality use and patient outcomes in palliative care: A scoping review [file sj-docx-2-dhj-10.1177_20552076231207574.docx]

**Supplementary File 2**

**VR review - papers excluded (n=20) at full text review**

Non-primary research (n = 16)

Wrong population (n = 1)

Wrong setting (n = 1)

Wrong context (n = 1)

Non-English Language (n = 1)

|  | **Title and Author** | **Notes** | **Final Decision** | **Reason** |
| --- | --- | --- | --- | --- |
|  | Amann and Blum (2021) ‘Digital Palliative Care’ | Abstract in English  Full text in German | Exclude | Non-English Language |
|  | Armstrong and Thomas (2021) ‘Incorporating Virtual Reality Into a Physical Exercise Programme for Patients With Parkinson’s Disease in an Outpatient Palliative Care Setting’. | Abstract only | Exclude | Non-primary research |
|  | Chuan (2020) ‘The use of Virtual Reality (VR) pain psychology therapy to relieve chronic pain associated with cancer’. | 2020 Cochrane abstract  Results not published | Exclude | Non-primary research |
|  | Blum (2021) ‘When digitalization goes viral, in the pandemic and in palliative care’. | Abstract only | Exclude | Non-primary research |
|  | Daubman *et al.* (2018)  ‘#PallTech: Leveraging Digital Resources in Hospice & Palliative Care’. | Conference abstract | Exclude | Non-primary research |
|  | Frydman et al 2020  Families of COVID-19 Patients Say Goodbye on Video:  A Structured Approach to Virtual End-of-Life Conversations | Letter to the Editor | Exclude | Non-primary research |
|  | Groninger, H. 2021  'I went to the beach without leaving my bed'--virtual reality for symptom management in advanced illness: Current evidence and applications’ | Literature review | Exclude | Non-primary research |
|  | Hsieh (2020) ‘Virtual reality video promotes effectiveness in advance care planning’ | Not palliative care for data collection | Exclude | Wrong setting |
|  | Hurd (2021) ‘Integration of Morphine and Virtual Reality: Pain Management for Adult Hospice Cancer Patients’ | Doctoral dissertation  Not published | Exclude | Non-primary research |
|  | Lee (2019) ‘The use of immersive virtual reality-would it be useful for the education in supportive care of cancer management?’ | Participants are students | Exclude | Wrong population |
|  | Lenz and Schmidt (2018) ‘Virtual Reality for Symptoms Control in Palliative Care’ | 2018 Cochrane abstract  Results not published | Exclude | Non-primary research |
|  | Groninger (2020) ‘Virtual Reality for Cancer Pain Management’ | 2020 Cochrane abstract  Results not published | Exclude | Non-primary research |
|  | Sichi (2020) ‘Look of Life 2.0. Virtual Reality for Cancer Patients in Home Palliative Care’ | 2021 Cochrane abstract  Results not published | Exclude | Non-primary research |
|  | Kim (2021) ‘Virtual Reality Therapy to Improve Physical and Psychological Symptoms and Quality of Life for End-of-life Patients on a Palliative Care Unit’ | 2021 Cochrane abstract  Results not published | Exclude | Non-primary research |
|  | Niki (2019) ‘The investigation for establishment of a new approach in palliative care using Virtual Reality’ | 2019 Cochrane abstract  (Niki 2019 with different title included in the review) | Exclude | Non-primary research |
|  | Payne *et al.* (2020)  ‘Digitisation and the patient–professional relationship in palliative care’ | Editorial | Exclude | Non-primary research |
|  | Taylor (2021) ‘The Room where it Happens’: An Exploration of Personalised Virtual Reality (VR) - Bringing the Home to the Hospice during the COVID-19 Pandemic’ | Abstract / Poster | Exclude | Non-primary research |
|  | Walker *et al.* (2017) ‘PATCH2 program: the creation of a virtual palliative care clinic’ | Not VR  Keeping in touch via tablets and smartphones, video conferencing | Exclude | Wrong context |
|  | Wang *et al.* (2020) ‘Virtual Reality as a Bridge in Palliative Care during COVID-19’ | Letter to the Editor | Exclude | Non-primary research |
|  | Weinstein Kaplan (2017)  ‘Virtual Reality Lets Patients Connect With the Outside  World or Explore a New One’ | Not primary research  Report / story | Exclude | Non-primary research |
